# Defined Natural Products as Adjunctive Therapies: Evidence-Gated Polypharmacology Across Molecular Targets, Human Exposure, Clinical Signals, and Safety Boundaries

**DOI:** 10.3390/ijms27167111

**Published:** 2026-08-08

**Authors:** Yohan Seo, Joohan Woo

**Affiliations:** 1 Department of Physiology, College of Medicine, Dongguk University, 123 Dongdae-ro, Gyeongju-si 38066, Republic of Korea; 2 Channelopathy Research Center (CRC), College of Medicine, Dongguk University, Goyang 10326, Republic of Korea

**Keywords:** defined natural products, polypharmacology, molecular pharmacology, adjunctive therapy, pharmacokinetics, target engagement, herb–drug interactions, curcumin, berberine, St John’s wort

## Abstract

Defined natural products are often framed as intrinsically safer multi-target alternatives to selective drugs, but this assumption is not pharmacologically justified. This critical review evaluates product-specific adjunctive evidence in respiratory allergy, inflammatory bowel disease, type 2 diabetes, depression, and insomnia. MEDLINE/PubMed records published from 2020 to 23 June 2026 were prioritized, while earlier pivotal randomized trials and quantitative safety studies were retained. Evidence was gated by product identity, human exposure, exposure-compatible target engagement, incremental efficacy, and safety boundaries. *Nigella sativa* oil improved Asthma Control Test scores by 1.5 points over placebo after 4 weeks, although the clinical importance is uncertain and FEV1 was not significantly improved. Curcumin plus mesalamine induced remission in mild-to-moderate ulcerative colitis (53.8% versus 0%), whereas indigo naturalis produced strong response rates but is constrained by pulmonary arterial hypertension. Berberine improves glycemic surrogates but lacks cardiovascular or renal outcome evidence and shows commercial potency variability. Saffron has a short-term antidepressant signal, while St John’s wort combines extract-dependent efficacy with CYP3A4/P-glycoprotein induction. Overall, selected defined natural products may merit narrow adjunctive roles, but undefined mixtures should not be treated as mechanistically established interventions without batch-level characterization, human pharmacokinetics, objective endpoints, and prospective interaction surveillance.

## 1. Introduction

Natural products remain major sources of chemical matter, pharmacological probes and therapeutic leads. Their contemporary value is not restricted to rediscovering single active molecules: chemically complex preparations can perturb several disease-relevant nodes at once, creating a testable form of polypharmacology. This property is attractive in chronic diseases whose phenotypes emerge from interacting epithelial, immune, metabolic, neural and microbial networks. It is nevertheless a property, not a verdict of clinical superiority [1,2,3,4].

Traditional preparations also present a translational problem that is more difficult than that of a single synthetic molecule. Species, plant part, cultivation, processing, extraction solvent, storage and adulteration can alter the composition of the administered product. Interactions among constituents can be additive, synergistic, antagonistic or simply irrelevant at human exposure. Accordingly, a tea, a decoction, a standardized extract and a purified phytochemical should be treated as different medicinal products even when they share a botanical source [5,6,7].

A second problem is target inflation. Database-driven network analyses can generate hundreds of predicted targets and enriched pathways, but these lists do not establish binding, intracellular exposure or causal target engagement. Curcumin is an instructive example: its broad in vitro reactivity and assay-interference properties can produce impressive pathway maps while obscuring whether unbound human concentrations reach the levels used in cell experiments. Network pharmacology therefore requires chemical and pharmacokinetic filters before biological interpretation [8,9,10,11].

Computational resources such as SwissADME, TCMSP and pathway databases are valuable for hypothesis generation, but their output should be followed by orthogonal assays, concentration–response relationships, human pharmacokinetics and disease-proximal biomarkers. In this review, a mechanism was considered clinically relevant only when the administered product was characterized, the direction of target modulation was consistent with the disease context, and the proposed effect was plausible at the observed or anticipated exposure [12,13,14].

Safety requires the same product-level specificity. Natural products can inhibit or induce CYP enzymes, UGTs and membrane transporters, alter coagulation or serotonergic tone, and produce idiosyncratic liver injury. These liabilities are especially important in chronic disease because polypharmacy is common and treatment duration is long. A favorable signal in a small efficacy trial cannot be extrapolated to patients receiving immunosuppressants, anticoagulants, oral contraceptives, antiretrovirals or narrow therapeutic index drugs without dedicated interaction data [15,16,17,18].

The objective of this review is narrower than the proposition that natural products overcome the toxicity of conventional medicines. We ask whether selected products provide incremental benefit when added to evidence-based care, whether their proposed mechanisms survive an identity-exposure-target-engagement audit, and whether their interaction and organ-toxicity boundaries are clinically manageable. The selected domains span airway inflammation, mucosal immunology, metabolic disease and neuropsychiatry, where multi-pathway claims are common but evidence quality is heterogeneous [19,20,21,22].

Against the broader resurgence of natural products in contemporary drug discovery, this review is deliberately product- and indication-specific rather than an encyclopedic catalog of botanicals [23,24]. The purpose is not to contrast “holistic” natural products with “single-target” drugs, but to test whether chemically defined natural products can sustain a molecularly interpretable chain from human exposure to target engagement, clinical signal, and manageable safety boundary.

## 2. Materials and Methods: Literature Strategy and Evidence Adjudication

### 2.1. Search Scope and Eligibility

This is a structured critical narrative review, not a registered systematic review. MEDLINE was searched through PubMed from database inception to 23 June 2026; records published from 1 January 2020 onward were prioritized. Earlier studies were retained when they were pivotal randomized trials, defined a quantitative pharmacokinetic or safety signal, or remained the only direct human evidence for a named preparation. Search blocks combined disease terms with the product, extract or phytochemical name and the terms randomized, placebo, meta-analysis, pharmacokinetic, toxicity, liver injury, pulmonary hypertension, interaction, CYP, or transporter. Reference lists of contemporary guidelines and major reviews were hand-searched to identify pivotal human studies. During final reference auditing, bibliographic records were checked against DOI-resolver, publisher, and PubMed metadata when available, and correction, erratum, expression-of-concern, and retraction status were screened. Legacy records without an independently confirmed DOI were retained only when the author, title, journal, year, volume, and pagination were stable and the record directly supported the cited claim. Products were selected purposively rather than exhaustively, to represent distinct outcomes across the five gates: a strong adjunctive efficacy signal with incomplete target engagement, a serious safety override, substantial product-potency variability, and an established clinical interaction liability. Inclusion required a named or reproducibly characterized preparation together with direct human efficacy, pharmacokinetic or quantitative safety evidence. This review is therefore an illustrative case selection and not a comprehensive botanical catalog. During preparation of this manuscript the authors used OpenAI ChatGPT (GPT-4o/GPT-5 class models, 2025–2026 releases) to assist with literature organization, language editing, reference-format auditing, and drafting of figure schematic concepts. Generative AI was not used to design the review, select or screen studies, extract or generate data, or draw scientific conclusions, and was not used to generate, fabricate or modify any scientific data. All quantitative values, chemical structures, figure labels and reference records were independently verified by the authors against the cited sources, and the authors take full responsibility for the content of this publication.

To align with IJMS scope, undefined crude mixtures and complex prescriptions were not treated as mechanistically established interventions; they were discussed only when a named commercial extract or a reproducible formula with disclosed ingredients, ratios, or marker-compound information was available.

### 2.2. Evidence Extraction and Adjudication

Evidence was extracted in a fixed sequence: product identity and formulation; population and disease state; design, comparator, dose and duration; validated endpoint; absolute and relative effect; statistical uncertainty; adverse events; interaction potential; and study limitations. Human randomized evidence was ranked above observational, animal and cellular evidence. Preclinical findings were used to explain a clinically observed signal or define a testable mechanism, not to declare therapeutic efficacy. Records were not used in the quantitative tables or in central clinical claims when the administered product could not be identified adequately, when the reported mechanism relied solely on computational target prediction, or when efficacy was inferred only from uncontrolled preclinical data. Mechanistic statements are labeled by evidence provenance throughout as cellular, animal-model, human pharmacokinetic, human biomarker, or clinical-outcome evidence, and preclinical findings are not presented as demonstrated human target engagement.

Clinical endpoints were ranked according to disease relevance. For asthma and rhinitis, ACT, exacerbations, FEV1, TNSS and medication use were preferred to isolated cytokines. For ulcerative colitis, clinical response was separated from clinical remission, endoscopic improvement, histologic improvement and relapse. For type 2 diabetes, HbA1c and FPG were treated as surrogate outcomes subordinate to cardiovascular, renal and mortality outcomes. For depression and insomnia, validated symptom scales, response, remission, relapse, functioning, actigraphy and polysomnography were distinguished from short-term self-reported well-being.

**Figure 1:**
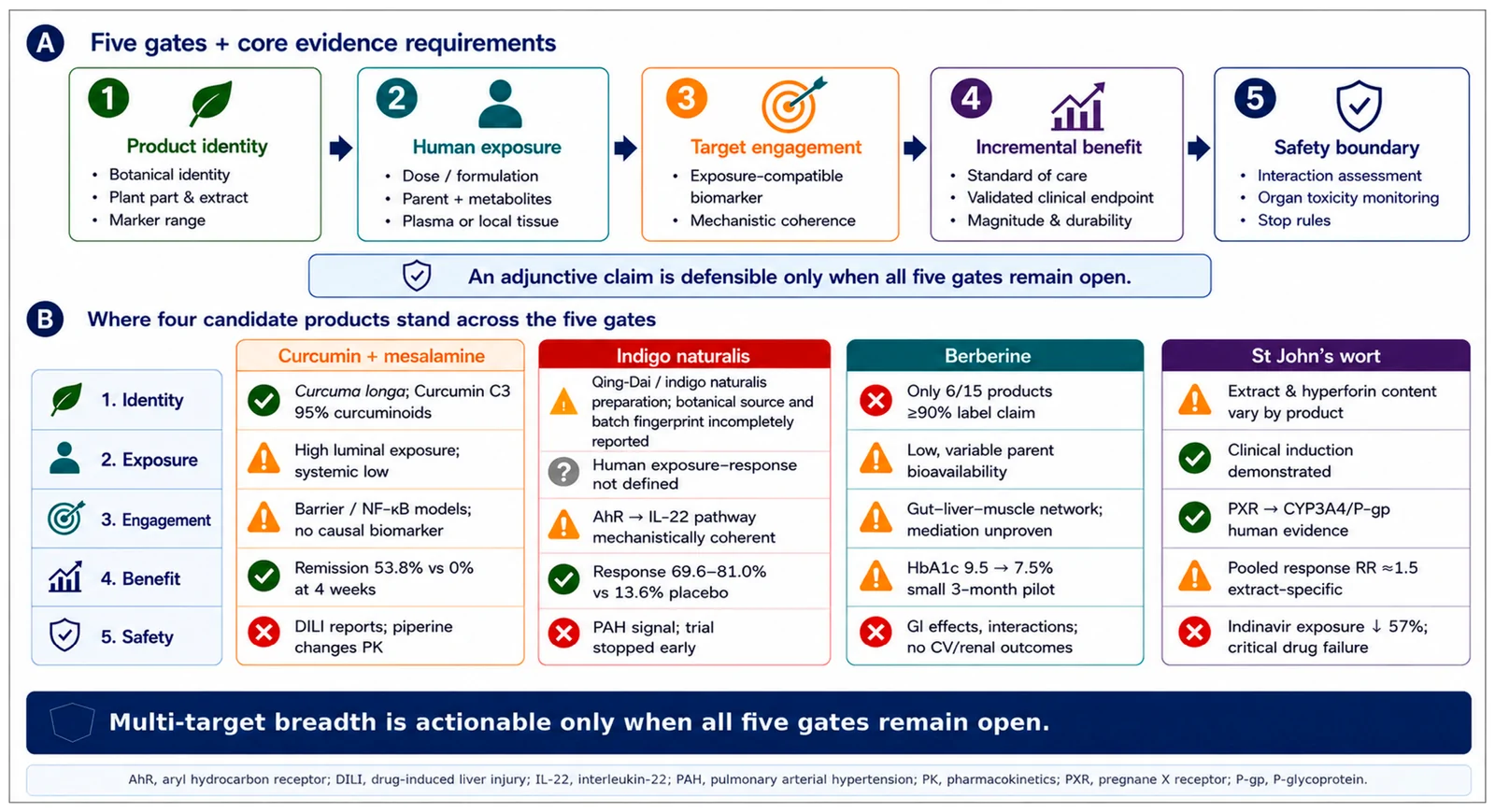
Evidence-gated development of defined natural products. (**A**) Five linked gates are required: authenticated product identity, human exposure, exposure-compatible target engagement, incremental clinical benefit and a defined safety boundary. (**B**) Four case studies illustrate distinct failure points. For curcumin, local luminal exposure is pharmacologically plausible but was not measured in the pivotal ulcerative-colitis RCT; enhanced-bioavailability formulations also introduce separate pharmacokinetic and liver-safety considerations. Indigo naturalis is constrained by pulmonary arterial hypertension (PAH), berberine by potency and exposure variability, and St John’s wort by pregnane X receptor (PXR)-mediated cytochrome P450 3A4 (CYP3A4) and P-glycoprotein (P-gp) induction. Color-coded status labels follow the rubric defined in Section 2.2 (green, directly supported by product-specific human evidence; amber, partial or incompletely characterized; red, not met or overridden by a clinically important safety limitation; question mark, insufficient information). They are qualitative author assessments, not formal GRADE certainty ratings, regulatory designations or clinical recommendations Colors, symbols and arrows are qualitative interpretive categories that distinguish evidence status and functional domains; they do not represent effect magnitude or any quantitative scale [25,26,27,28,29,30,31,32,33,34,35,36,37,38,39,40,41].

No class-wide safety assumption was permitted. Evidence was adjudicated through five linked gates: authenticated product identity, plausible human exposure, exposure-compatible target engagement, incremental benefit over contemporary standard care, and a defined safety or interaction boundary. A serious replicated safety signal could override an efficacy signal. Source-reported event counts, percentages, effect estimates, confidence intervals and *p* values were transcribed without inferential reanalysis. No new confidence interval, risk difference or pooled estimate was calculated by the authors. Quantitative tabulation was restricted to pivotal human trials and established meta-analyses that directly informed the product- and indication-specific evidence assessment. Figure 1 summarizes the framework, and Table 1, Table 2 and Table 3 provide the quantitative efficacy, product/exposure and safety evidence used in the narrative. Status labels in Figure 1 are qualitative author assessments and are not formal GRADE ratings or regulatory designations. They were applied using a fixed rubric: green, the criterion is directly supported by product-specific human evidence; amber, the evidence is partial, indirect or incompletely characterized; red, the criterion is not met or is overridden by a clinically important safety limitation; and a question mark, information is insufficient for assessment.

**Table 1:** Representative quantitative human efficacy evidence for selected natural products. All values are source-reported; heterogeneous products and endpoints preclude direct ranking across rows.

Product/Indication	Design and Analysis Population	Intervention (Dose, Duration)	Primary Endpoint	Key Quantitative Findings	Interpretation and Principal Limitation	Ref.
*Nigella sativa* oil/partly controlled asthma	Double-blind placebo RCT; 80 randomized, 60 completed	500 mg twice daily; 4 weeks	ACT at week 4	ACT at week 4: 21.1 ± 2.6 vs. 19.6 ± 3.7 (*p* = 0.044). Eosinophil change: −50 vs. +15 cells/µL (*p* = 0.013). FEV1 change: +4 vs. +1 percentage points (*p* = 0.170).	Patient-reported control and eosinophils improved, but the 1.5-point ACT difference has uncertain clinical importance; the airflow endpoint was not significant and follow-up was short.	[42]
Curcumin + mesalamine/active mild-to-moderate UC	Double-blind placebo RCT; 50 randomized, 41 completed	Curcumin 3 g/day; 4 weeks	Clinical remission at week 4	Clinical remission 14/26 vs. 0/24 (53.8% vs. 0%). Clinical response 17/26 vs. 3/24 (65.3% vs. 12.5%). Endoscopic remission ~38% vs. 0%.	Large absolute effects, but the trial was small, short and formulation-specific.	[25]
Curcumin + 5-ASA/UC maintenance	Multicenter double-blind placebo RCT; 89 randomized	Curcumin 1 g twice daily; 6 months	Relapse at 6 months	Relapse 2/43 vs. 8/39 (4.7% vs. 20.5%); source *p* = 0.049.	Clinically relevant maintenance signal; few events, legacy endpoint framework and no equivalent-scale replication.	[26]
Indigo naturalis/active UC	Multicenter dose-ranging placebo RCT; *n* = 86 randomized	0.5, 1.0 or 2.0 g/day; 8 weeks	Clinical response at week 8	Clinical response 69.6%, 75.0%, 81.0% vs. 13.6%; remission 13.0%, 55.0%, 38.1% vs. 4.5%; mucosal healing 56.5%, 60.0%, 47.6% vs. 13.6%.	Strong clinical-response signal, but remission and mucosal healing were non-monotonic across doses. Small treatment arms and early termination after an external PAH signal preclude identification of an optimal dose.	[27]
*Boswellia serrata*/Crohn’s disease remission maintenance	Double-blind placebo RCT; 108 enrolled, 82 randomized (42/40), 66 analyzed	Standardized extract; 52 weeks	Remission maintenance at 52 weeks	Remission maintained 59.9% vs. 55.3% (*p* = 0.85); no statistically persuasive superiority despite acceptable tolerability.	High-value negative trial; mechanism and pilot data did not translate to the primary clinical endpoint.	[43]
Berberine/newly diagnosed type 2 diabetes	Small active-comparator pilot	0.5 g three times daily; 3 months	HbA1c and fasting plasma glucose at 3 months	Within berberine arm: HbA1c 9.5 ± 0.5% to 7.5 ± 0.4%; FPG 10.6 ± 0.9 to 6.9 ± 0.5 mmol/L; postprandial glucose 19.8 ± 1.7 to 11.1 ± 0.9 mmol/L.	Within-arm metabolic activity only; the pilot does not establish non-inferiority to metformin and provides no CV-renal outcomes.	[34]
Berberine/type 2 diabetes	Systematic review and meta-analysis; 46 RCTs, 4158 participants	Variable products and regimens	Pooled glycemic and lipid outcomes	Pooled signals favored berberine for glucose, insulin resistance and lipids.	High heterogeneity, geographic concentration and variable trial quality limit certainty.	[35]
Cinnamon/type 2 diabetes or prediabetes	Meta-analysis and meta-regression; 16 studies, 1098 participants	Species, dose and duration varied	Pooled fasting glucose and HbA1c	Fasting glucose and insulin-resistance measures generally favored cinnamon; HbA1c findings were inconsistent.	Species and coumarin content were often incompletely controlled; pooled estimates were heterogeneous.	[44]
St John’s wort/major depression	Systematic review; 35 studies, approximately 6993 participants	Standardized extracts; duration varied	Pooled treatment response	Versus placebo: response RR 1.53 (95% CI 1.19–1.97; I^2^ = 79%; 18 RCTs, N = 2922). Comparative efficacy was broadly similar to antidepressants in pooled analyses.	Extract-dependent evidence with substantial heterogeneity; rare interactions and treatment failures are not captured adequately by efficacy RCTs.	[37]
Saffron/depression and anxiety	Meta-analysis; 23 studies	Typically ~30 mg/day; mostly 6–12 weeks	Pooled depression symptom score	Depression vs. placebo: Hedges’ g = 0.99 (*p* < 0.001); anxiety g = 0.95 (*p* = 0.006); adjunctive depression g = 1.23 (*p* = 0.028).	Large short-term effects with small-study, regional and publication-bias concerns.	[45]

ACT, Asthma Control Test; CV, cardiovascular; FEV1, forced expiratory volume in 1 s; FPG, fasting plasma glucose; PAH, pulmonary arterial hypertension; RCT, randomized controlled trial; RR, risk ratio; UC, ulcerative colitis; 5-ASA, 5-aminosalicylate. All values in this table are source-reported; no effect estimate or confidence interval was calculated by the authors.

**Table 2:** Product identity, marker-compound control, human exposure and target-engagement matrix for the studied preparations.

Product/Studied Preparation	Identity/Marker-Compound and Batch Control	Human Exposure Evidence	Target-Engagement Evidence	Principal Translational Gap	Ref.
Petasin-standardized butterbur (Ze 339)	Defined Ze 339 extract standardized to petasin/isopetasin-related markers; independent pyrrolizidine-alkaloid removal and batch testing are essential. Raw herb, tea and unverified supplements are not equivalent.	Human pharmacokinetics have not been linked prospectively to rhinitis response.	Leukotriene and eosinophil functional data are mechanistically coherent, but in-trial target engagement was not established.	Independent replication, HPLC/LC-MS batch comparability, PA testing and product-specific benefit–risk assessment.	[46,47,48,49,50,51,52]
*Nigella sativa* oil, 500 mg twice daily	Seed-oil preparation; thymoquinone or other marker-compound ranges, oxidation state and pressing/extraction conditions should be specified. Trial-dose oil is not equivalent to black-seed tea or culinary exposure.	No trial-linked parent/metabolite pharmacokinetic dataset.	ACT and eosinophil signals were observed; no validated pathway biomarker or significant FEV1 effect.	Standardized chemistry, controller-therapy background, exacerbation endpoints and longer follow-up.	[42,53,54,55]
Curcumin capsules used in UC trials	Trial-grade curcumin capsules should disclose curcuminoid content, dissolution characteristics and enhancer status. The 3 g/day induction and 1 g twice-daily maintenance products are not interchangeable with culinary turmeric or piperine-enhanced products.	Local intestinal exposure is pharmacologically plausible but was not measured in the pivotal RCTs; piperine materially changes systemic exposure.	NF-κB, inflammasome and barrier hypotheses remain unconfirmed by prospective mucosal target-engagement markers.	Mucosal PK/PD, centralized endoscopy/histology, formulation reproducibility, HPLC fingerprinting and liver-safety stopping rules.	[8,25,26,32,33,56,57,58,59]
Indigo naturalis (Qing-Dai), 0.5–2.0 g/day	Multicomponent Qing-Dai preparation containing indigo/indirubin-related constituents; source material, processing, contaminant testing and batch fingerprints should be disclosed. Active constituents and exposure–response remain incompletely resolved.	A clinically interpretable human exposure–response relationship has not been established.	AhR–IL-22 signaling is coherent with epithelial repair, but mediation of the clinical effect has not been demonstrated.	Active-constituent definition, duration-dependent PAH risk modeling and prospective cardiopulmonary monitoring.	[27,28,29,30,31,60]
*Boswellia serrata* standardized extract	Formulation-specific resin extract; boswellic-acid profile, extraction solvent and batch comparability should be specified. Findings cannot be generalized across unstandardized commercial *Boswellia* products.	Boswellic-acid exposure was not linked to the durable clinical endpoint.	A 5-lipoxygenase/leukotriene rationale did not translate into Crohn’s remission maintenance.	Product-specific PK/PD and clinically meaningful replication rather than mechanistic extrapolation.	[43,61,62]
Berberine, commonly 0.5 g three times daily	Berberine-containing alkaloid products require identity, salt form, purity, dissolution and label-claim verification; only 6 of 15 tested US products contained ≥90% of labeled content.	Low and variable parent exposure; metabolites and local gut effects may dominate.	AMPK, gut–liver–muscle and microbiome mechanisms are predominantly preclinical and not causally resolved in humans.	Population PK, CYP/transporter interaction studies, standardized batches and cardiovascular–renal outcome trials.	[34,35,36,63,64,65,66,67,68,69,70,71,72,73]
Cinnamon preparations	Species-defined *Cinnamomum* preparation is essential; cassia and *C. verum* differ in coumarin exposure. Plant part, extract type, coumarin content and marker compounds should be specified.	Active-constituent exposure varies; concentrated cassia products may exceed the coumarin TDI.	Insulin-sensitivity hypotheses have not been anchored consistently to proximal human biomarkers.	Species-defined, composition-controlled trials with hepatic safety monitoring.	[44,74,75,76]
St John’s wort standardized extracts	*Hypericum* extracts should declare hyperforin and hypericin content; low- and high-hyperforin products are not interchangeable because hyperforin largely determines interaction liability.	Human induction has delayed onset/offset and cannot be managed by separating administration times.	PXR activation with CYP3A4 and P-gp induction is demonstrated in humans.	Efficacy and interaction findings must remain extract-specific; high-risk co-medication often precludes use.	[17,37,38,39,40,41,77,78,79,80,81,82]
Standardized saffron extracts, typically ~30 mg/day	*Crocus sativus* stigma extracts should declare crocin, crocetin, safranal and/or picrocrocin ranges; culinary saffron is not dose-equivalent to standardized trial extracts.	Human parent/metabolite and brain-exposure data remain limited.	Monoamine, neuroplasticity and inflammatory mechanisms are largely hypothesis-generating.	Independent multicenter trials, functional recovery, relapse prevention and rare-event surveillance.	[45,83,84,85,86,87,88,89]
*Ziziphus*-containing formulas	Formula-level evidence should disclose species, plant parts, ingredient ratios, extraction method and active-marker ranges; most clinical formulas remain incompletely standardized.	No defined active-constituent PK framework for most clinical formulations.	GABAergic and related mechanisms remain predominantly preclinical; objective sleep mediation is unproven.	Credible placebo matching, standardized formula, actigraphy/polysomnography and next-day safety endpoints.	[90,91,92]

ACT, Asthma Control Test; AhR, aryl hydrocarbon receptor; FEV1, forced expiratory volume in 1 s; PA, pyrrolizidine alkaloid; PAH, pulmonary arterial hypertension; PK/PD, pharmacokinetics/pharmacodynamics; P-gp, P-glycoprotein; PXR, pregnane X receptor; TDI, tolerable daily intake; UC, ulcerative colitis.

**Table 3:** Safety, toxicity and herb–drug interaction matrix for clinical decision-making and trial monitoring.

Product	Documented or Plausible Hazard	Quantitative/Clinical Signal	High-Risk Context	Risk Mitigation and Monitoring	Ref.
Butterbur	Pyrrolizidine-alkaloid hepatotoxicity, genotoxicity and vascular injury	Risk is linked to PA exposure; hepatobiliary events have also prompted scrutiny of manufactured extracts.	Unverified raw herb, tea or supplements; liver disease; concomitant hepatotoxins.	Use only independently verified PA-free standardized product; avoid raw preparations; investigate liver symptoms promptly.	[51,52]
*Nigella sativa*	GI symptoms, allergy and uncertain interaction profile at concentrated doses	Four-week asthma RCT did not establish long-term organ safety.	Pregnancy, polypharmacy, anticoagulant or glucose-lowering treatment without monitoring.	Use trial-matched product; monitor symptoms and relevant co-medications; do not substitute for controller therapy.	[42,54]
Curcumin/turmeric	GI effects, bleeding concern, idiosyncratic liver injury; altered exposure with enhancers	Piperine markedly increases systemic exposure (Section 5.5); DILIN and case reports describe clinically important liver injury.	Enhanced-bioavailability products; liver disease; anticoagulants; multiple supplements.	Specify formulation; baseline and symptom-triggered liver tests in long trials; interaction review; stop for jaundice or marked enzyme elevation.	[32,33,59]
Indigo naturalis	Pulmonary arterial hypertension, hepatic injury and other cardiopulmonary events	The UC RCT was terminated early after an external PAH signal; a Japanese nationwide survey identified 11 PAH cases among 877 users and also recorded liver dysfunction, intussusception, and gastrointestinal events.	Chronic use, dyspnea, syncope, cardiopulmonary disease, unsupervised consumer use.	Not recommended for unsupervised use; baseline and serial cardiopulmonary assessment in research, liver monitoring, and immediate discontinuation/evaluation for dyspnea, syncope, or edema.	[27,28,29,30]
*Boswellia*	GI symptoms and uncertain product purity; limited long-term database	52-week Crohn’s disease trial reported acceptable tolerability but no efficacy advantage.	Use as substitute for effective IBD therapy; poorly characterized extracts.	Do not infer class efficacy; verify product and monitor disease activity objectively.	[43]
Berberine	GI intolerance, altered microbiota, CYP/transporter interactions; pregnancy and neonatal concerns	GI adverse effects were common in early studies; commercial products show marked potency variability (Table 2).	Pregnancy, breastfeeding, infants, polypharmacy, narrow therapeutic index drugs.	Use standardized product; titration and GI monitoring; medication reconciliation; avoid in pregnancy/infancy pending safety evidence.	[34,36,67]
Cassia cinnamon	Coumarin-related hepatotoxicity and additive glucose lowering	Coumarin TDI approximately 0.1 mg/kg/day; content varies substantially by species and product.	Liver disease, high-dose chronic use, combination with hepatotoxic or glucose-lowering drugs.	Identify species and coumarin content; avoid concentrated chronic dosing when composition is unknown.	[75,76]
St John’s wort	CYP3A4/P-gp induction, serotonergic toxicity, contraceptive failure and therapeutic failure of critical drugs	Indinavir exposure fell ~57%; reduced contraceptive-hormone exposure and breakthrough bleeding were documented, with transplant rejection and digoxin interactions also reported.	Transplantation, HIV, oncology, oral contraception, anticoagulation, concomitant serotonergic drugs.	Generally avoid in high-risk co-medication; review hyperforin content; allow for delayed onset/offset of induction; monitor drug concentrations where appropriate.	[17,38,39,40]
Saffron	GI or neurologic symptoms; limited high-dose and pregnancy safety data	Most depression trials used ~30 mg/day for 6–12 weeks; rare-event power was low.	Pregnancy, bleeding risk, high-dose nonstandard preparations, severe psychiatric disease.	Use standardized trial-range dose; do not delay urgent psychiatric care; collect long-term safety and recurrence data.	[45,83,85]
*Ziziphus*/sedative formulas	Sedation, psychomotor impairment and additive CNS depression	Meta-analyses rely mainly on subjective outcomes; no robust objective sleep-effect estimate or next-day performance dataset is available.	Older adults, falls risk, driving, alcohol, benzodiazepines or other sedatives.	Assess next-day function and falls; avoid unmonitored sedative combinations; prioritize CBT-I.	[90,92,93]

CBT-I, cognitive behavioral therapy for insomnia; CNS, central nervous system; CYP3A4, cytochrome P450 3A4; DILIN, Drug-Induced Liver Injury Network; GI, gastrointestinal; PA, pyrrolizidine alkaloid; PAH, pulmonary arterial hypertension; P-gp, P-glycoprotein; TDI, tolerable daily intake.

## 3. From Multi-Target Activity to Interpretable Molecular Pharmacology

Multi-target pharmacology can arise through two distinct architectures. A single molecule can bind several targets, or multiple constituents can contribute separate activities. The first architecture can often be studied with conventional concentration–response and target-deconvolution methods. The second requires quantitative chemical profiling, bioavailability data and mixture-aware pharmacology because a nominal botanical dose does not reveal the concentration of each active constituent. Failure to distinguish these architectures encourages the assumption that every predicted component contributes to efficacy [3,4,6].

The translational chain begins with identity. Voucher specimens, genetic authentication where appropriate, plant-part specification, extraction solvent, extraction yield, HPLC/LC-MS or equivalent chromatographic fingerprints, quantitative marker-compound ranges and contaminant testing should be reported. For multi-herb formulas, each ingredient, plant part, ratio and marker range should be disclosed. Batch-level comparability is not a cosmetic quality-control exercise: it determines whether a trial can be reproduced and whether a commercial product is pharmacologically equivalent to the investigational material [5,7].

Exposure is the second gate. Parent phytochemicals may be poorly absorbed, rapidly conjugated or transformed by gut microorganisms, while local luminal concentrations can exceed plasma concentrations by orders of magnitude. This distinction is central to curcumin in UC and to berberine in metabolic disease. A low systemic bioavailability does not automatically invalidate a local intestinal mechanism, but it does invalidate claims that require sustained free concentrations in distant organs unless metabolites or tissue accumulation are demonstrated [22,94,95].

Target engagement should be proximal and prespecified. A change in C-reactive protein after eight weeks is not proof that an extract inhibited a specific kinase, whereas a dose-dependent change in a validated pathway biomarker that correlates with clinical response is more informative. Omics can identify responsive modules, but post hoc enrichment should be separated from prospective pharmacodynamic hypotheses. This distinction reduces the risk of reverse-engineering a mechanism around a positive clinical outcome [10,11,12].

Clinical benefit must be incremental. A credible adjunctive trial compares standard care plus the natural product with the same standard care plus matched placebo, preserves blinding, specifies rescue medication and reports both absolute effects and uncertainty. Comparisons against outdated or subtherapeutic conventional regimens are not sufficient. For strongly flavored or scented products, sensory matching and assessment of treatment-guess accuracy should be considered because broken blinding can inflate patient-reported outcomes.

Finally, the safety boundary must be tested rather than inferred. Liver chemistry, renal function, electrocardiography or disease-specific monitoring should reflect the expected toxicology. Medication reconciliation should include non-prescription products. Interaction studies should be prioritized when constituents activate nuclear receptors, inhibit conjugating enzymes or alter transporters. The clinical relevance of this framework becomes apparent in St John’s wort, where efficacy and interaction liability are inseparable, and in indigo naturalis, where a strong UC efficacy signal coexists with a rare but serious pulmonary vascular hazard [15,17,18,19].

## 4. Respiratory Allergic Disease

### 4.1. Contemporary Standard Care and the Adjunctive Space

Asthma and allergic rhinitis arise from barrier dysfunction, innate alarmins, type 2 lymphoid and T-helper responses, IgE-dependent mast-cell activation, eosinophilic inflammation and neural or structural changes. These pathways support a network view of disease, but they also explain why selective biologics can be highly effective: targeting IgE, IL-5, the IL-4 receptor alpha chain or upstream epithelial cytokines can interrupt pivotal network nodes. Multi-target breadth should therefore be compared with actual disease control, not with a caricature of one-receptor pharmacology [96,97,98].

Current rhinitis guidance supports intranasal corticosteroids, second-generation antihistamines, combination intranasal therapy and allergen immunotherapy according to phenotype and severity. Asthma guidance requires ICS-containing treatment and emphasizes reduction in exacerbation risk. First-generation antihistamine sedation and systemic corticosteroid toxicity are legitimate clinical concerns, but they do not apply uniformly to modern non-sedating antihistamines or appropriately dosed intranasal and inhaled corticosteroids. Natural products should be tested as add-ons in patients whose baseline care is optimized [99,100,101,102].

### 4.2. Butterbur: Standardized Extract Evidence Cannot Be Generalized to Raw Herb

The petasin-standardized butterbur extract Ze 339 has been evaluated in seasonal allergic rhinitis. A 125-participant randomized comparison with cetirizine and subsequent placebo- and antihistamine-controlled studies reported symptom improvement, with a lower sedative burden than the cetirizine comparator in the early trial. Petasin-related inhibition of leukotriene biosynthesis and eosinophil effector function provides a coherent anti-inflammatory rationale, although manufacturer involvement, short duration and limited replication constrain confidence [46,47,48,49].

The safety conclusion is product-specific. Butterbur naturally contains pyrrolizidine alkaloids capable of hepatic sinusoidal injury, genotoxicity and carcinogenicity. Only products with validated PA removal and batch testing resemble the studied extract. Raw butterbur, unverified supplements and home-prepared tea cannot be considered equivalent; reports of hepatobiliary events also show that a ‘PA-free’ label does not eliminate the need to investigate manufacturing quality, concomitant drugs and causality [50,51,52].

### 4.3. Nigella sativa: A Measurable Asthma-Control Signal with Limited Physiological Confirmation

In a double-blind trial, 80 adults with partly controlled asthma were randomized to *Nigella sativa* oil 500 mg twice daily or placebo for four weeks; 60 completed the study. At week 4, ACT was 21.1 ± 2.6 with *Nigella* and 19.6 ± 3.7 with placebo (between-group difference 1.5 points; *p* = 0.044). The change in blood eosinophils was −50 cells/µL (interquartile range −155 to −1) versus +15 cells/µL (−60 to 87; *p* = 0.013). By contrast, the median change in percent-predicted FEV1 was +4 versus +1 percentage points and was not significant (*p* = 0.170) [42].

Thymoquinone and other seed constituents modulate oxidative and inflammatory signaling in experimental systems, and clinical reviews identify signals across asthma and metabolic outcomes. The asthma trial nevertheless shows why patient-reported and physiological endpoints must be separated: a 1.5-point ACT difference and eosinophil change over four weeks do not establish exacerbation prevention, corticosteroid-sparing efficacy or altered airway remodeling. Replication should quantify thymoquinone content, document adherence and ICS background therapy, and include longer exacerbation follow-up [53,54,55].

### 4.4. Multi-Herb Formulas and Non-Pharmacological Comparators

Chinese herbal formulas have produced mixed rhinitis and asthma signals in small trials: some seasonal-rhinitis formulas improved symptoms whereas others showed no benefit over placebo, and the anti-asthma herbal medicine intervention produced early positive signals. However, formula composition, diagnostic stratification, background therapy and placebo matching differ across studies. Ephedra-containing prescriptions add sympathomimetic cardiovascular risk, glycyrrhizin-containing prescriptions can cause hypokalemia and pseudoaldosteronism, and *Aristolochia* contamination must be excluded analytically. These issues preclude class-wide claims for Xiao-Qing-Long-Tang or related formulas [103,104,105,106].

Saline nasal irrigation is a useful benchmark because it is inexpensive, mechanistically simple and supported by a Cochrane review showing possible improvement in symptoms and quality of life, albeit with low-certainty evidence. It does not test the multi-target hypothesis, but it illustrates that adjunctive value can arise without systemic pharmacology. Sterile, distilled or previously boiled water is essential. For an orally administered natural product to justify greater interaction and quality-control burden, it should outperform this kind of low-risk supportive intervention on validated outcomes [107,108].

**Figure 2:**
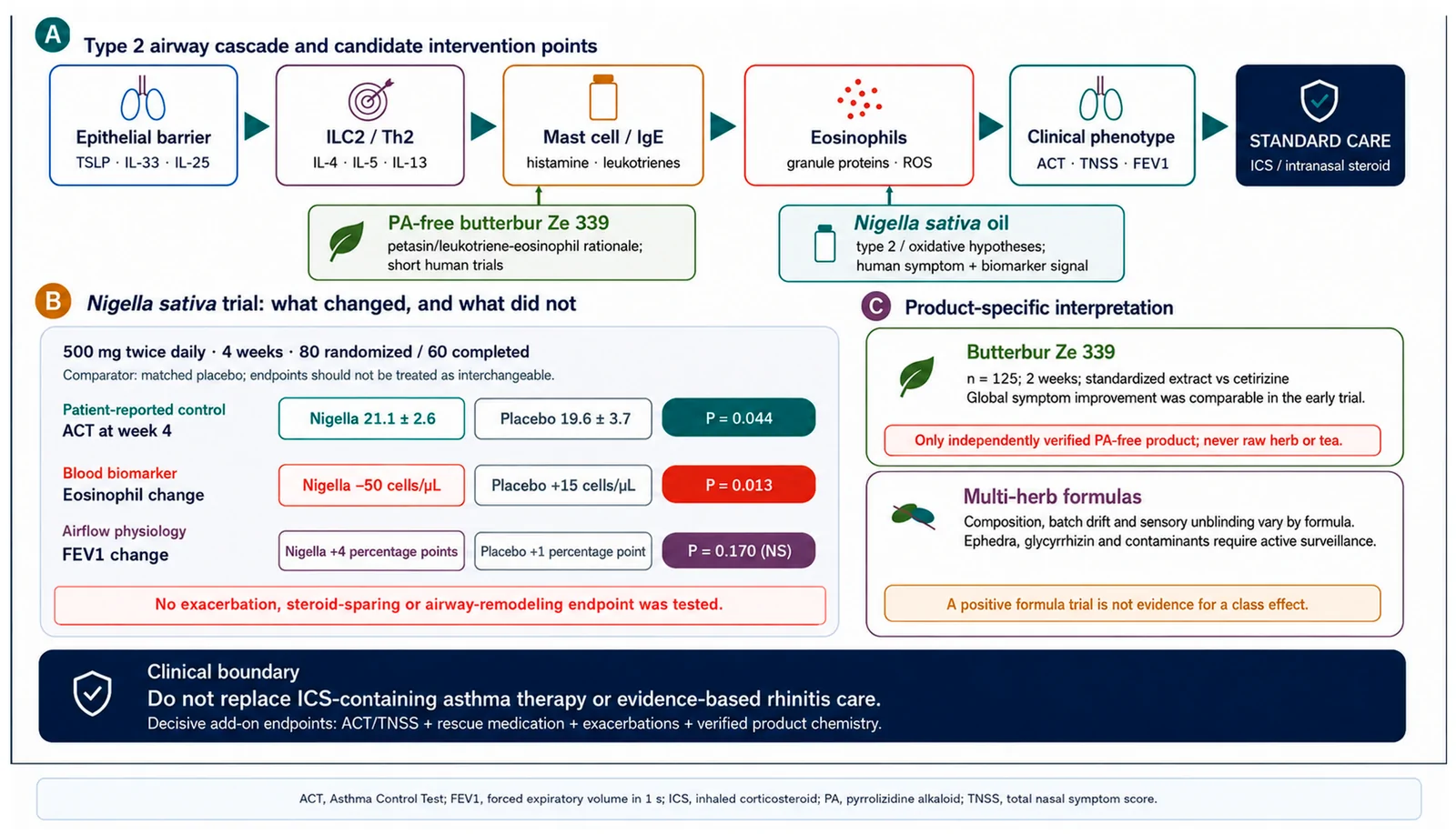
Respiratory allergy: mechanism, quantitative trial signal and clinical boundary. (**A**) Candidate products are mapped to points in the type 2 airway cascade as hypotheses rather than proven causal targets. (**B**) In the 4-week *Nigella sativa* trial, the Asthma Control Test (ACT) and blood eosinophil count improved, whereas the forced expiratory volume in 1 s (FEV1) did not change significantly; quantitative values are given in Table 1. The ACT difference reached statistical significance, but its clinical importance is not established. (**C**) Butterbur evidence applies only to the defined, independently verified pyrrolizidine-alkaloid (PA)-free Ze 339 preparation and represents a short-term symptom signal rather than a class effect. Multi-herb formulas require formula-specific evaluation. None replaces inhaled corticosteroid (ICS)-containing asthma therapy or evidence-based rhinitis care. Colors, symbols and arrows are qualitative interpretive categories that distinguish evidence status and functional domains; they do not represent effect magnitude or any quantitative scale. TNSS, total nasal symptom score [42,46,47,48,49,50,51,52,99,100,101,102,103,104,105,106,107,108].

Overall, respiratory evidence supports a restricted hierarchy. PA-free Ze 339 and standardized *Nigella* oil are plausible research candidates; saline irrigation is a pragmatic adjunct; and heterogeneous multi-herb preparations require product-specific trials. None should replace ICS-containing therapy in asthma or intranasal corticosteroid-based care in persistent rhinitis. The main research opportunity is not wholesale substitution but measurement of incremental symptom control, rescue-medication reduction and exacerbation prevention under contemporary standard care [109,110]. The pathway-level evidence and product-specific boundaries are summarized in Figure 2.

## 5. Inflammatory Bowel Disease

### 5.1. Why the Intestine Is a Distinctive Target Organ

IBD combines epithelial barrier failure, innate and adaptive immune activation, microbial dysbiosis and altered stromal or vascular responses. Current UC and Crohn’s disease treatment uses 5-aminosalicylates, corticosteroids, immunomodulators, biologics and small molecules according to severity and prognostic risk. Treat-to-target frameworks distinguish symptom control from endoscopic healing and increasingly incorporate biomarkers. This distinction is critical for natural-product studies, which often improve symptoms without proving durable modification of mucosal inflammation [111,112,113,114].

**Figure 3:**
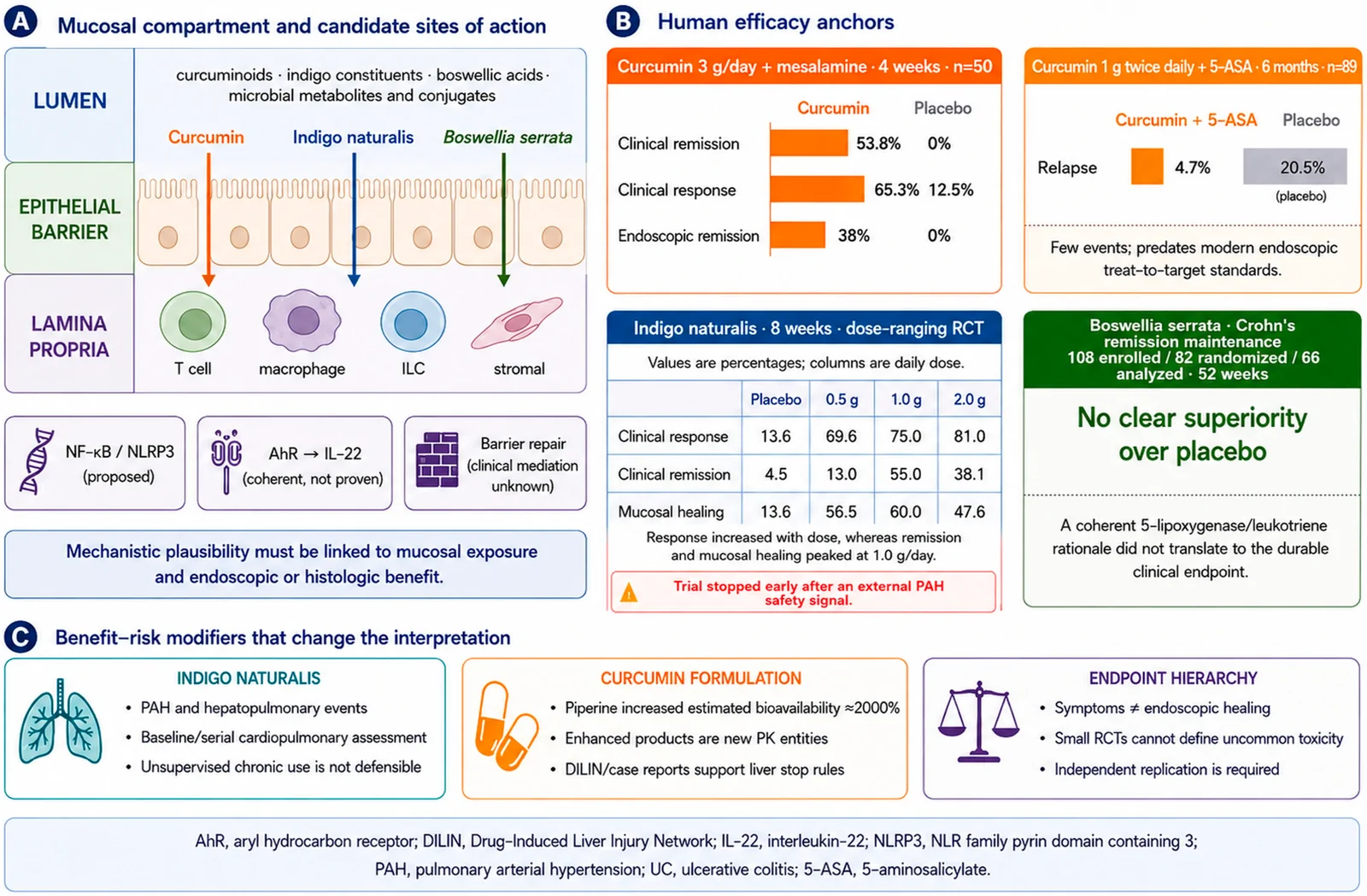
Intestinal barrier pharmacology: compartment, efficacy and safety. (**A**) Curcumin, indigo naturalis and *Boswellia* are positioned in proposed luminal, epithelial and immune modules; quantitative trial values are given in Table 1. (**B**) In the indigo naturalis panel, equal-size markers show percentages and do not represent statistical weights. The RCT was stopped early after an external PAH signal. A subsequent nationwide survey reported 11 PAH cases among 877 users; this survey proportion is not a prospective incidence estimate. *Boswellia* did not clearly maintain Crohn’s disease remission (108 enrolled, 82 randomized, 66 analyzed). Clinical response to indigo naturalis increased with dose, whereas remission and mucosal healing peaked at 1.0 g/day; this non-monotonic pattern is unstable and does not identify an optimal dose (Section 5.3). Microbiome compositional change does not establish causal mediation. (**C**) Benefit–risk modifiers that change the interpretation. Colors, symbols and arrows are qualitative interpretive categories that distinguish evidence status and functional domains; they do not represent effect magnitude or any quantitative scale. 5-ASA, 5-aminosalicylate; AhR, aryl hydrocarbon receptor; DILIN, Drug-Induced Liver Injury Network; IL-22, interleukin-22; UC, ulcerative colitis [25,26,27,28,29,30,31,32,33,43].

The gastrointestinal tract also offers a pharmacological advantage: poorly absorbed compounds can reach high luminal concentrations and interact directly with epithelial, immune and microbial compartments. This makes UC a more plausible indication for some low-bioavailability products than systemic inflammatory disease. The same local exposure can produce toxicity; however, microbial transformation can generate unmeasured metabolites. Product composition, stool or mucosal pharmacokinetics and endoscopic outcomes should therefore be integrated [95,115,116]. Figure 3 integrates mucosal mechanisms with the divergent clinical and safety signals.

### 5.2. Curcumin as an Adjunct to Mesalamine

The most persuasive efficacy trial enrolled 50 patients with active mild-to-moderate ulcerative colitis despite mesalamine and randomized them to curcumin 3 g/day or placebo for four weeks; 41 patients completed treatment. Clinical remission occurred in 14/26 versus 0/24 participants (53.8% versus 0%), and clinical response occurred in 17/26 versus 3/24 (65.3% versus 12.5%). Endoscopic remission was reported in approximately 38% versus 0%. The effects are large, but precision and external validity remain limited by the small sample, short follow-up and single formulation [25].

A separate maintenance trial randomized 89 patients in remission to curcumin 1 g twice daily or placebo in addition to sulfasalazine or mesalamine. At six months, relapse occurred in 2/43 evaluable curcumin recipients and 8/39 placebo recipients (4.7% versus 20.5%; source-reported *p* = 0.049). This clinically relevant signal addresses maintenance rather than symptom fluctuation, but the trial predates contemporary endoscopic targets, contains few events and has not been replicated at equivalent scale [26,117].

Meta-analytic summaries generally favor curcumin for induction or maintenance when added to standard therapy, but they combine different formulations, doses, disease activity criteria and endpoints. Small-study effects and publication bias remain plausible. A robust confirmatory program would use centralized endoscopic reading, fecal calprotectin, histology, prespecified rescue criteria and an extract with quantified curcuminoids and dissolution characteristics. It should also establish whether benefit persists when modern optimized mesalamine dosing is used [113,118].

### 5.3. Indigo Naturalis: High Efficacy Signal, Narrow Safety Margin

In a multicenter dose-ranging RCT of active UC, 86 participants were assigned to placebo (*n* = 22) or indigo naturalis 0.5 g/day (*n* = 23), 1.0 g/day (*n* = 20), or 2.0 g/day (*n* = 21) for eight weeks. The prespecified primary endpoint was clinical response at week 8, which was 13.6% with placebo and 69.6%, 75.0% and 81.0% with ascending doses. Clinical remission, a secondary endpoint, was 4.5% with placebo and 13.0%, 55.0% and 38.1% with ascending doses, and mucosal healing was 13.6% with placebo and 56.5%, 60.0% and 47.6% with ascending doses. These results are stronger than most botanical trials and support a genuine pharmacological effect. The clinical signal is mechanistically consistent with preclinical AhR-IL-22 and epithelial-repair findings, but pathway mediation and target engagement were not demonstrated in trial participants [27,60].

Although clinical response increased across the three doses, neither clinical remission nor mucosal healing showed a monotonic dose–response pattern: both peaked at 1.0 g/day and were lower at 2.0 g/day. The published report does not establish that this attenuation resulted from high-dose intolerance or differential dropout. The trial was terminated after an external report of pulmonary arterial hypertension in a person who had taken self-purchased indigo naturalis for six months, rather than after a dose-specific toxicity signal within the 2.0 g/day group, and no serious adverse event was recorded during the trial. With approximately twenty participants per arm, a difference in a few events changes these secondary-endpoint proportions substantially, and enrollment stopped before the planned sample was reached. The non-monotonic pattern should therefore be read as unstable and hypothesis-generating rather than as evidence that 1.0 g/day is an optimal dose or that receptor-level engagement is lost at higher exposure. It also reinforces the exposure gap identified in Table 2: without a defined active constituent and a human exposure–response model, no dose of indigo naturalis can be specified as optimal [27,28,60].

The trial was terminated early after an external PAH safety signal. A subsequent Japanese nationwide survey of 877 users identified 11 PAH cases, together with liver dysfunction and gastrointestinal adverse events, while systematic and prospective evidence confirmed that toxicity is product- and duration-dependent. PAH may improve after discontinuation, but reversibility does not make the event acceptable for unsupervised chronic use. Baseline cardiopulmonary assessment, symptom surveillance, liver chemistry and product-level impurity characterization are essential in research; general consumer use cannot be recommended on current evidence [27,28,29,30,31].

### 5.4. Boswellia and the Value of Negative Evidence

*Boswellia serrata* resin contains boswellic acids with 5-lipoxygenase-related and other anti-inflammatory activities. Early small studies in UC and active Crohn’s disease generated interest, but a 52-week maintenance trial in Crohn’s disease—108 patients enrolled, of whom 82 were randomized (42 to Boswelan, 40 to placebo) before the study was stopped early for futility, and 66 were analyzable—found a good safety profile without clear efficacy over placebo (remission maintained in 59.9% versus 55.3%; *p* = 0.85). This result is methodologically valuable: a coherent leukotriene mechanism and positive pilot studies did not predict success on a clinically relevant maintenance endpoint [43,61,62].

### 5.5. Bioavailability Engineering Can Change Both Efficacy and Interaction Risk

Curcumin has low and variable oral systemic exposure because of poor dissolution, metabolism and conjugation. Piperine 20 mg administered with 2 g curcumin increased estimated bioavailability by approximately 2000% in a small human pharmacokinetic experiment. That finding is often presented as a formulation solution, but piperine can also alter intestinal metabolism and transport. Enhanced-bioavailability products should therefore be treated as new pharmacological entities rather than interchangeable versions of conventional curcumin [32,56,57,58].

Safety surveillance has also changed the risk assessment. Turmeric and curcumin are generally well tolerated in trials, yet case reports and a Drug-Induced Liver Injury Network series describe clinically important hepatotoxicity, including products designed for enhanced absorption. The absolute risk is uncertain, but the signal is sufficient to justify stopping rules and liver testing in long trials, particularly when symptoms such as pruritus, jaundice or dark urine appear [18,33,59].

Microbiome modulation is frequently invoked to explain botanical efficacy in IBD. The concept is biologically plausible because epithelial cells, microbial metabolites and host immunity are tightly coupled, and multi-omics studies demonstrate disease-associated ecological disruption. Nevertheless, compositional changes after treatment do not establish mediation. Causal inference requires longitudinal sampling, metabolite measurement and a demonstration that microbiome changes precede and statistically account for clinical or endoscopic improvement [119,120,121,122].

Fecal microbiota transplantation trials show that manipulating the intestinal ecosystem can induce remission in a subset of UC patients, but protocols, donor effects and safety requirements are substantial. These data support the gut ecosystem as a therapeutic compartment; they do not validate every product described as ‘microbiome restoring’. Natural-product studies should avoid interpreting an increase in a nominally beneficial genus as proof of disease modification [123,124].

## 6. Type 2 Diabetes and Metabolic Syndrome

### 6.1. The Comparator Has Changed

Contemporary type 2 diabetes treatment is organized around glycemic control, weight, hypoglycemia risk and cardiovascular, renal and hepatic outcomes. Metformin remains widely used, while SGLT2 inhibitors and GLP-1 receptor agonists have outcome-based roles in defined patients. A botanical that reduces FPG over several weeks cannot be described as equivalent to a drug class supported by cardiovascular or renal outcome trials. Its defensible initial claim is an effect on surrogate metabolic endpoints [125,126].

The pathophysiology includes hepatic glucose production, muscle insulin resistance, adipose lipolysis, altered incretin biology, beta-cell dysfunction, renal glucose handling and central regulation. This ‘ominous octet’ makes multi-organ pharmacology plausible, but conventional drugs are not necessarily mono-organ agents: metformin, for example, affects hepatic energetics, gut signaling and other pathways. The scientific comparison should therefore be between quantified incremental benefits and risks, not between ‘single-target synthetic’ and ‘holistic natural’ labels [127,128,129].

### 6.2. Berberine: Consistent Surrogate Signals, Incomplete Translational Control

In study A of an early three-month report, 36 participants with newly diagnosed type 2 diabetes were randomized to berberine 0.5 g three times daily or metformin. In the berberine arm, HbA1c fell from 9.5 ± 0.5% to 7.5 ± 0.4%, FPG from 10.6 ± 0.9 to 6.9 ± 0.5 mmol/L, postprandial glucose from 19.8 ± 1.7 to 11.1 ± 0.9 mmol/L, and triglycerides from 1.13 ± 0.13 to 0.89 ± 0.03 mmol/L. These within-arm changes demonstrate metabolic activity, but the small, short study was not designed or powered to establish non-inferiority to metformin and provides no cardiovascular or renal outcome evidence [34].

A second study reported improvements in glycemia and lipids with berberine, and subsequent meta-analyses expanded the evidence base. A 2021 synthesis included 46 randomized trials and 4158 participants and found favorable signals for glucose, insulin resistance and lipid parameters. Interpretation is limited by heterogeneous background therapy, variable product quality, regional concentration of trials, short duration and risk-of-bias concerns. Effect estimates are therefore more credible as proof of metabolic activity than as a basis for replacing guideline-directed therapy [35,63,64,65].

Product inconsistency is not theoretical. Substantial commercial potency variability has been documented in tested US preparations (Table 2). Thus, two patients taking the same nominal dose can receive substantially different exposure. Trials should report identity, alkaloid content, dissolution and batch data, and clinical recommendations should refer to the tested product rather than to ‘berberine’ as an undifferentiated commodity [36].

**Figure 4:**
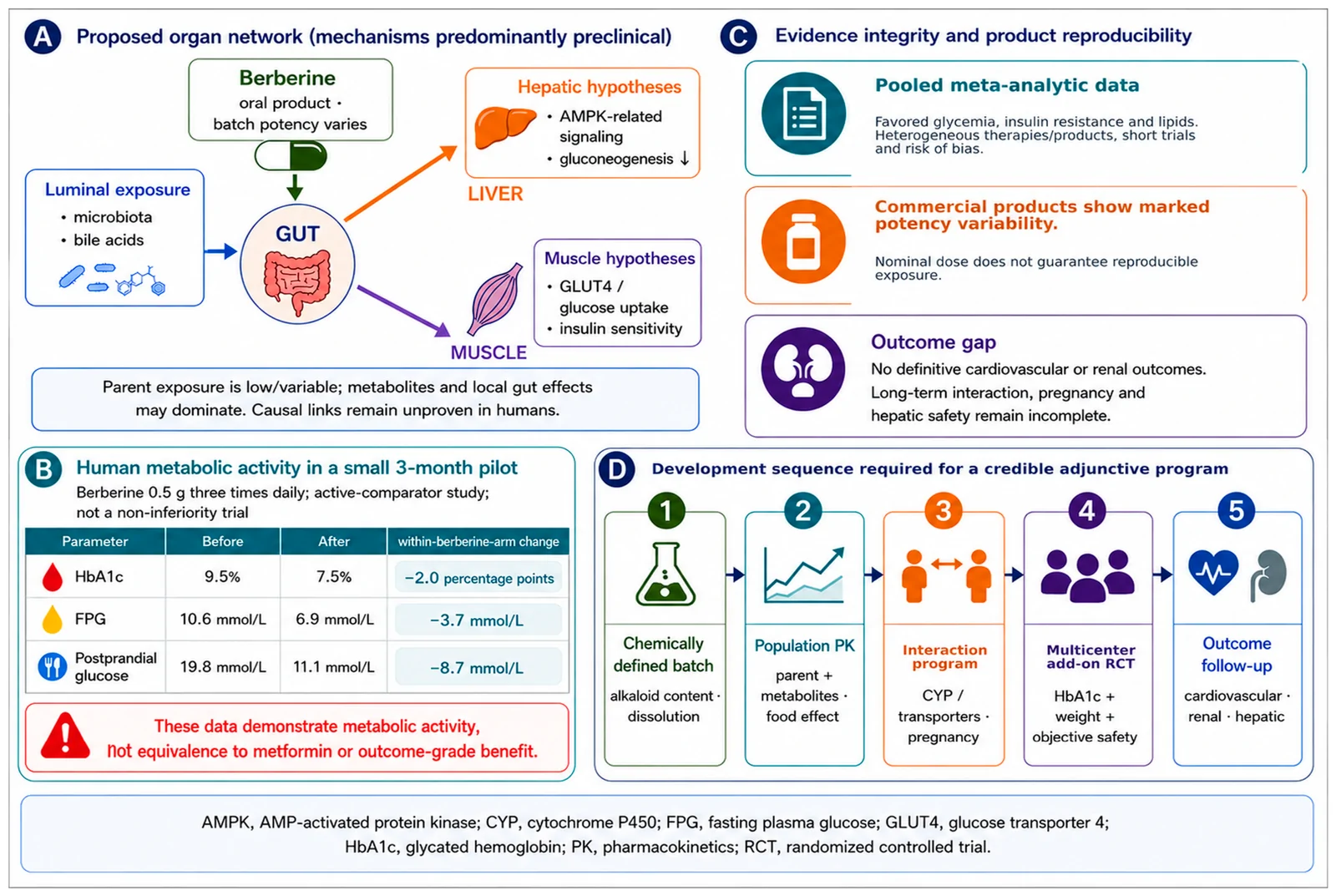
Berberine: metabolic signal and translational gaps. (**A**) Proposed gut–liver–muscle mechanisms are predominantly preclinical. (**B**) The displayed values are within-berberine-arm changes in glycated hemoglobin (HbA1c), fasting plasma glucose (FPG) and postprandial glucose in a small 3-month active-comparator report; quantitative values are given in Table 1. These values are not the between-group treatment difference, do not establish non-inferiority to metformin and do not demonstrate outcome-grade benefit. (**C**) Pooled meta-analytic data support metabolic-surrogate activity, whereas commercial preparations show marked potency variability (Table 2). (**D**) Development requires chemically defined batches, population pharmacokinetics (PK), cytochrome P450 (CYP) and transporter interaction testing, multicenter add-on RCTs and cardiovascular (CV), renal and hepatic outcome follow-up Colors, symbols and arrows are qualitative interpretive categories that distinguish evidence status and functional domains; they do not represent effect magnitude or any quantitative scale [34,35,36,66,67,68,69].

Proposed mechanisms involving AMPK, hepatic gluconeogenesis, GLUT4 trafficking, lipid metabolism, bile acids, gut microorganisms and inflammatory pathways are supported predominantly by cellular and animal-model evidence and have not been causally linked to the human glycemic signal. These findings are useful for selecting biomarkers, but the low oral bioavailability and extensive intestinal handling of berberine complicate attribution to a single systemic target. Changes in microbial composition can be consequences of antimicrobial exposure, altered diet or gastrointestinal adverse effects rather than mediators of improved insulin sensitivity [66,67,68,69]. Figure 4 summarizes the proposed gut–liver–muscle network and the principal translational gaps.

Recent reviews continue to identify berberine as a candidate for metabolic and cardiovascular indications, but many proposed effects originate from animal models or concentrations not linked to human tissue exposure. The immediate clinical-development priority is a standardized formulation with population pharmacokinetics, defined metabolites, interaction studies and a multicenter add-on trial. Long-term cardiovascular, renal, hepatic and pregnancy safety require dedicated evidence [70,71,72,73].

### 6.3. Cinnamon and Other Food-Derived Interventions

Meta-analyses of cinnamon trials report reductions in FPG and some indices of insulin resistance, but estimates are heterogeneous and HbA1c effects are inconsistent. Species matters: cassia cinnamon generally contains more coumarin than *Cinnamomum verum*. The tolerable daily intake for coumarin is approximately 0.1 mg/kg body weight, and habitual high-dose use can exceed this threshold depending on product composition. Food familiarity therefore does not guarantee pharmacological safety at concentrated doses [44,74,75,76].

Inflammation and the gut microbiota link obesity to insulin resistance, providing a mechanistic foundation for diet- and microbiome-directed interventions. Yet human metabolic phenotypes are influenced by medication, adiposity, sleep, diet and socioeconomic context, and microbiome associations are population-specific. Natural-product trials should stratify or adjust for these factors and should not present a microbial signature as a universal biomarker without external validation [130,131,132,133].

Human microbiome studies support bidirectional relationships between metabolic disease and intestinal ecology, but causal intervention data remain less mature than observational maps. Future berberine studies should measure antimicrobial-resistance determinants, bile-acid pools and gastrointestinal symptoms alongside glycemia. This would distinguish beneficial metabolic reprogramming from nonspecific perturbation of the intestinal ecosystem [134,135].

## 7. Mild-to-Moderate Depression and Insomnia

### 7.1. Standard Treatment, Discontinuation and the Danger of False Equivalence

Major depressive disorder is heterogeneous and can involve monoaminergic, inflammatory, neuroendocrine, circadian and neuroplastic mechanisms. Antidepressants have modest average efficacy but clear value for many patients, and psychotherapy is a core treatment. Adverse effects, sexual dysfunction and withdrawal symptoms are clinically important, yet they do not justify unsupervised substitution in severe depression, suicidality, psychosis or bipolar-spectrum illness [136,137].

Antidepressant discontinuation symptoms can include dizziness, sensory disturbances, anxiety, sleep disruption, nausea and flu-like symptoms. Hyperbolic tapering has been proposed to reduce large changes in serotonin-transporter occupancy at low doses, while guideline-based management emphasizes gradual, individualized dose reduction and monitoring. No natural product has been established as a pharmacological ‘bridge’ that reliably prevents SSRI withdrawal, and adding serotonergic botanicals can complicate diagnosis or increase toxicity [138,139,140].

### 7.2. St John’s Wort: Efficacy Is Inseparable from Extract and Interaction Profile

Systematic reviews support efficacy of some standardized St John’s wort extracts for some patients with mild-to-moderate depression. Across 18 placebo-controlled RCTs (N = 2922), the pooled responder risk ratio was 1.53 (95% CI 1.19–1.97; I^2^ = 79%), while comparative analyses generally found response similar to conventional antidepressants and fewer adverse-event discontinuations. The evidence is not uniform across products, hyperforin content, disease severity or trial setting. Large US trials failed to demonstrate superiority over placebo in major or minor depression, and continuation data do not establish relapse prevention across formulations. Efficacy should therefore be attributed to the tested extract, not to the plant category [37,77,78,79].

The *Hypericum* Depression Trial and a later minor-depression trial illustrate the negative evidence: neither the botanical intervention nor the active antidepressant comparator clearly separated from placebo under the studied conditions. Such findings can reflect population selection, assay sensitivity or genuine lack of effect. They argue against presenting St John’s wort as a universally effective natural SSRI and support product-specific, severity-specific interpretation [80,81,82].

Hyperforin activates pregnane X receptor and induces CYP3A4 and P-gp. In a clinical study, St John’s wort reduced indinavir exposure by approximately 57%, a magnitude capable of causing therapeutic failure. Interactions have also been documented with oral contraceptives, transplant immunosuppressants and digoxin. Because induction develops and resolves over time, risk persists beyond a single co-administered dose and cannot be managed by simply separating administration times [38,39,40,41].

This interaction profile changes the clinical position of the product. Medication reconciliation must include prescription drugs, non-prescription products and planned procedures. Patients receiving calcineurin inhibitors, antiretrovirals, selected anticancer therapies, oral contraceptives or other critical CYP3A/P-gp substrates should generally avoid hyperforin-rich St John’s wort. Concomitant serotonergic therapy also raises a pharmacodynamic concern. A low-hyperforin extract may have a different interaction profile, but that does not permit extrapolation from efficacy trials of other preparations [17,21].

### 7.3. Saffron: Promising Short-Term Efficacy with Geographic and Small-Study Limitations

A 2019 meta-analysis of 23 studies reported large placebo-controlled effects on depressive symptoms (Hedges’ g = 0.99; *p* < 0.001) and anxiety symptoms (g = 0.95; *p* = 0.006), with an adjunctive antidepressant effect of g = 1.23 (*p* = 0.028). These estimates are clinically interesting but probably optimistic because many trials were small, short, and concentrated within a limited number of research networks; publication-bias analyses also raised concern. The effect size should be interpreted as a signal requiring independent replication rather than a stable estimate of routine-care benefit [45].

Subsequent systematic reviews and a 2025 comparison with SSRIs continued to support short-term efficacy and tolerability, but did not resolve long-term recurrence, functional recovery or rare adverse events. Standardized saffron extracts commonly deliver approximately 30 mg/day, whereas culinary use does not reproduce the studied dose or chemical profile. Future trials should quantify crocin, crocetin and safranal, use independent multicenter recruitment and include remission and relapse endpoints [83,84,85].

An add-on trial of a standardized saffron extract in adults with persistent symptoms despite antidepressant medication provides a clinically relevant design because it tests incremental rather than replacement therapy. Earlier pilot comparisons with fluoxetine or imipramine generated similar symptom improvement, but small sample size and assay sensitivity prevent claims of equivalence. Animal antidepressant-like effects of crocin and crocetin support mechanism generation, not clinical efficacy [86,87,88,89].

### 7.4. Insomnia: Subjective Improvement Is Not Sleep-Architecture Normalization

Chinese herbal formulas containing *Ziziphus spinosa* seed and valerian preparations have been evaluated for insomnia. Meta-analyses often report improvement in Pittsburgh Sleep Quality Index scores, but allocation concealment, blinding, placebo matching and publication bias are recurrent limitations. Objective measures such as actigraphy or polysomnography, next-day psychomotor performance and rebound insomnia are sparse. The evidence therefore supports low-certainty symptomatic benefit, not a demonstrated normalization of sleep architecture [90,91,92].

**Figure 5:**
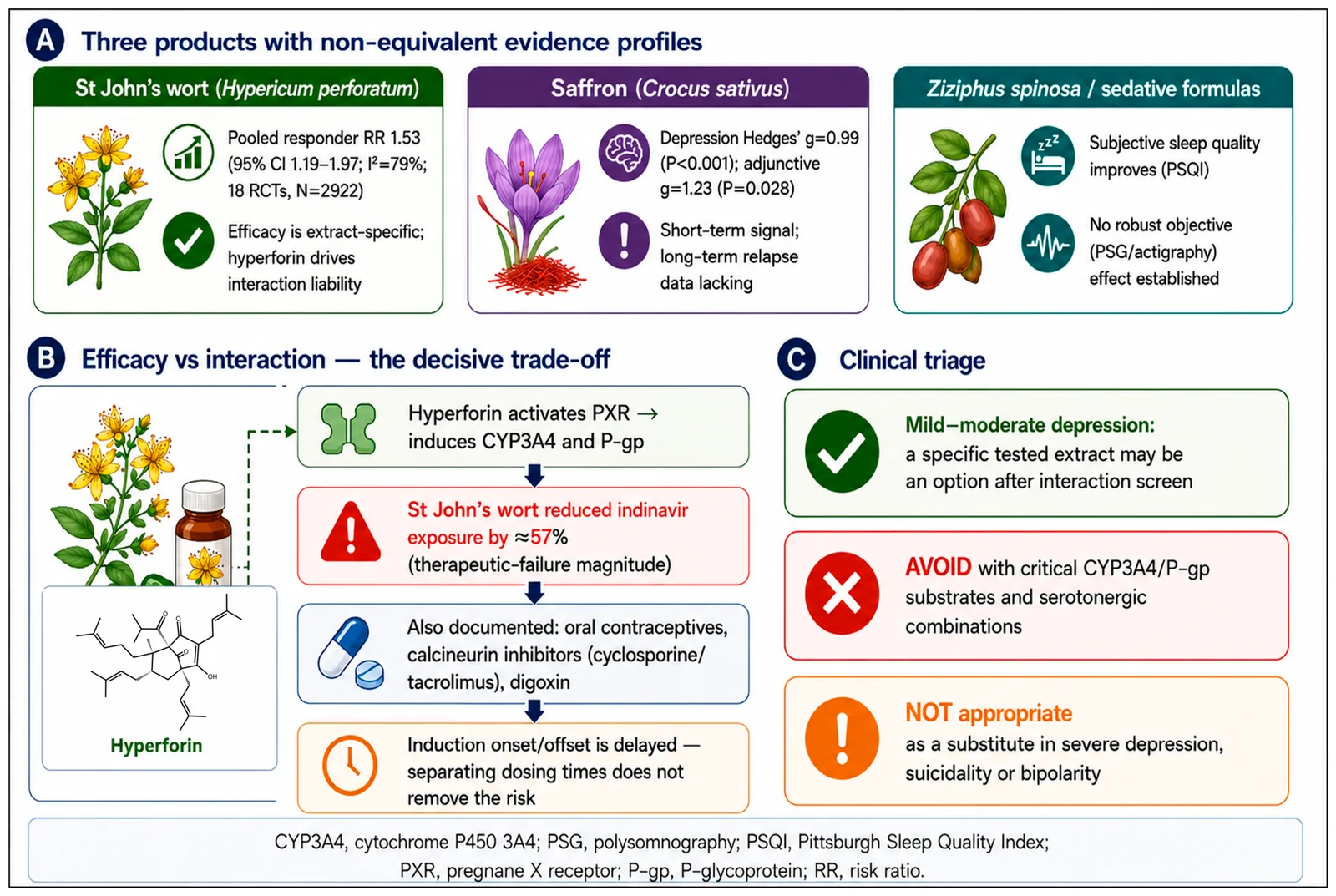
Neuropsychiatric phytotherapy: efficacy, interaction and clinical triage. (**A**) Saffron has a short-term antidepressant signal, but small-study, regional and publication-bias concerns limit stability of the estimate. For St John’s wort, the pooled placebo-controlled response favored the extract, while negative US trials and extract dependence constrain generalization. (**B**) Hyperforin-rich extracts activate PXR and induce CYP3A4 and P-gp; clinically important reductions in the exposure of critical substrates, together with breakthrough bleeding, transplant rejection and digoxin interactions, have been reported (Table 3). Efficacy is extract-specific, whereas interaction liability is determined by hyperforin content. Pooled estimates are given in Table 1. *Ziziphus*-containing formulas rely mainly on subjective sleep outcomes, with no robust objective effect estimate. (**C**) Urgency screening, medication reconciliation, optimization of standard care, product eligibility and prespecified efficacy and harm endpoints should precede clinical use or study. Colors, symbols and arrows are qualitative interpretive categories that distinguish evidence status and functional domains; they do not represent effect magnitude or any quantitative scale. CBT-I, cognitive behavioral therapy for insomnia; MDD, major depressive disorder [37,38,39,40,41,45,80,81,82,83,84,85,86,90,91,92,93].

Gut–brain signaling, inflammatory activation and sleep loss interact bidirectionally, offering multiple mechanistic entry points for natural products. However, these broad networks can make almost any anti-inflammatory or microbiome change appear relevant to mood or sleep. Translational credibility requires mediation analysis that connects exposure to a proximal biomarker and then to a prespecified clinical outcome, while accounting for expectancy, sedative co-medication and baseline circadian behavior [141,142,143,144]. Figure 5 contrasts the efficacy, interaction and evidence profiles of the principal neuropsychiatric candidates.

The 2023 European insomnia guideline emphasizes cognitive behavioral therapy for insomnia as first-line treatment and evidence-based pharmacotherapy when indicated. Natural products should be compared with this standard, not with untreated chronic insomnia. A pragmatic adjunctive trial could test whether a chemically defined product improves sleep efficiency and daytime function after cognitive behavioral therapy has been offered, with prospective monitoring for sedation, falls and interaction effects [93].

## 8. Cross-Cutting Interpretation: Where the Evidence Converges

Across the four disease domains, no product satisfies all five evidence gates. Curcumin in ulcerative colitis is the clearest adjunctive signal because the product is delivered to the diseased compartment and was tested with mesalamine using clinical and endoscopic endpoints. *Nigella* oil and saffron have measurable short-term symptom signals but no hard long-term outcomes. Berberine has repeated metabolic-surrogate signals but weak control of product potency and exposure. St John’s wort has clinically relevant efficacy in selected extracts and an interaction liability that can dominate its benefit.

The review also identifies three recurring inferential errors. First, a multi-pathway diagram is treated as proof of multi-target efficacy. Second, a statistically significant change in a surrogate is presented as disease modification. Third, a favorable average adverse-event profile is generalized to patients with complex co-medication. These errors can be reduced by predefining the product, causal pathway, clinical endpoint and interaction surveillance before the trial begins.

Effect size should be interpreted with sample size and event count. Curcumin’s 53.8-percentage-point remission difference and indigo naturalis response rates above 69% are striking, yet both derive from trials too small to characterize uncommon toxicity or treatment-effect heterogeneity. Saffron’s large standardized mean effects are similarly vulnerable to small-study and publication biases. Replication by independent groups is a pharmacological requirement, not merely a statistical preference.

Negative evidence is equally informative. The absence of a significant FEV1 change in the *Nigella* trial, the lack of clear *Boswellia* efficacy in Crohn’s disease remission maintenance, and placebo-sensitive St John’s wort trials prevent selective citation of positive outcomes. A high-quality review should treat these findings as constraints on the claim, identify design explanations without dismissing the result, and specify what a decisive next experiment would measure.

Product standardization repeatedly changes the conclusion. PA-free Ze 339 is not equivalent to butterbur tea; enhanced-bioavailability curcumin is not equivalent to culinary turmeric; berberine products with large potency differences cannot share one dose–response relationship; and St John’s wort extracts with different hyperforin content do not share one interaction profile. Regulatory and clinical language should name the exact preparation whenever possible.

The term ‘dose-sparing’ also requires direct evidence. A reduction in background medication is beneficial only if disease control, exacerbations and long-term outcomes are preserved. None of the reviewed products currently justifies routine reduction in ICS, biologic therapy, glucose-lowering therapy or antidepressants without a structured protocol. Dose sparing should be a randomized endpoint with rescue rules, not an inference from improved symptoms.

Economic arguments deserve similar discipline. Botanical products can be inexpensive at purchase but costly when quality testing, clinical monitoring, interactions and treatment failure are included. Conversely, a standardized low-cost adjunct that prevents relapse could be economically valuable. Cost-effectiveness studies should therefore use verified products and measure total health care utilization, not retail price alone.

Patient preference remains important. Many patients use teas, supplements or traditional formulas without informing clinicians. A nonjudgmental medication history can prevent interaction harm and creates an opportunity to distinguish culinary exposure from pharmacological dosing. Shared decision-making should communicate the strength of evidence, the exact product studied, the limits of substitution and the symptoms that require discontinuation or urgent evaluation.

## 9. A Translational Development Agenda

### 9.1. Product and Analytical Standards

Each clinical batch should have botanical authentication, contaminant screening, extraction yield, chromatographic fingerprint and quantitative marker ranges. Multi-herb formulas need ingredient-level traceability and tests for substitutions or undeclared pharmaceuticals. Stability and dissolution should be established for the study duration. These data should be published in the article or an accessible supplement rather than held only in a manufacturer certificate [5,7].

### 9.2. Human Pharmacokinetics and Target Engagement

Phase 1 studies should characterize parent compounds, active metabolites, food effects and between-person variability, including the contribution of the gut microbiome when relevant. Sampling should match the proposed site of action: plasma alone may be inadequate for a luminal UC therapy, while stool, mucosal biopsy or imaging may be justified. Target engagement should be selected before efficacy analysis and should be compatible with measured unbound exposure [22,94].

### 9.3. Clinical Trial Design

Trials should use add-on designs against current standard care, centralized randomization, credible sensory matching and prespecified rescue medication. Disease-specific core outcomes should be reported with absolute effects, confidence intervals and missing-data handling. Adaptive enrichment may be reasonable when a product is expected to work in a biomarker-defined phenotype, but subgroup claims require interaction testing rather than within-group significance.

Sample size should reflect both efficacy and safety. Botanical trials are often powered for a symptom score over four to eight weeks and are therefore incapable of excluding clinically important uncommon toxicity. Extension studies, registries or distributed pharmacovigilance can complement RCTs, provided product identity is retained. Pooling adverse events across chemically different products should be avoided.

### 9.4. Herb–Drug Interaction Programs

Interaction risk can be triaged using constituent concentration, in vitro inhibition or induction and clinical exposure margins. Products with plausible PXR, constitutive androstane receptor, CYP, UGT or transporter effects should undergo appropriately designed probe-substrate studies. Pharmacodynamic interactions with anticoagulants, glucose-lowering agents, sedatives and serotonergic drugs require separate evaluation. Real-world medication lists should guide prioritization [15,16,20,21].

### 9.5. Biomarkers and Causal Inference

Biomarkers should serve a defined purpose: exposure, target engagement, response prediction or safety. Broad cytokine panels and untargeted microbiome analyses are exploratory unless linked to a prespecified causal model. Longitudinal mediation analysis, replication cohorts and negative controls can distinguish a treatment mediator from a correlate of disease improvement. Single-cell and spatial methods are particularly useful when they identify the cell population engaged at clinically achieved exposure.

### 9.6. Independent Replication and Reporting

Trials should be registered before recruitment, report the full chemical specification, publish protocols and statistical analysis plans, and disclose manufacturer roles. Null and negative results must remain visible. Independent replication is especially important when early effects are large, samples are small or most studies arise from one country or research network. Data and code sharing would allow reanalysis of endpoint definitions and missing-data assumptions.

### 9.7. Computational, Multi-Omic and Precision Approaches

Several emerging technologies are reshaping how natural-product hypotheses are generated, but none of them replaces a gate. Machine learning models now support structure elucidation, constituent prioritization and bioactivity prediction across natural-product chemical space, and can rank candidates faster than phenotypic screening alone [145]. Their output is a ranked hypothesis rather than evidence of human target engagement, and bias toward well-characterized scaffolds in the training data remains a practical constraint. Network pharmacology and network toxicology extend the same logic to mixtures and to predicted liabilities; systematic comparison of predicted with experimentally verified targets shows useful concordance for some pathways, but the value of a prediction still depends on subsequent confirmation at a concentration that is achievable in humans [146,147]. Computational breadth that is not constrained by quantitative product chemistry and clinically achieved exposure reproduces the target-inflation problem described in Section 3 at greater scale.

The technologies with the most direct effect on the gates are analytical and quantitative. Untargeted metabolomics and mass-spectrometry imaging can convert batch identity from a certificate statement into a measured chemical fingerprint and can localize constituents within tissue, which addresses product authentication and part of the exposure gate [148]. Quantitative systems pharmacology and model-informed development provide a formal way to link measured exposure, predicted target occupancy and a clinical endpoint, which is the inference that most botanical trials currently leave implicit [149]. Quantitative proteomics of drug-metabolizing enzymes and transporters supports both interaction prediction and patient stratification, and is directly relevant to products such as St John’s wort whose liability is determined by induction of a defined enzyme-transporter pair [150].

Precision-medicine framing should therefore be specific rather than aspirational. For the products reviewed here, the realistic near-term stratification variables are enzyme and transporter genotype or phenotype for interaction risk, baseline endoscopic or inflammatory activity for intestinal products, measured product potency for berberine, and baseline microbial or metabolite signatures for compounds such as curcumin and berberine whose conversion to active metabolites depends on the intestinal microbiota. Each of these is testable within an add-on trial. All of them require prespecified hypotheses, external validation and prospective clinical confirmation, and none substitutes for product authentication, human pharmacokinetics or causal target-engagement studies.

## 10. Limitations of This Review

This review used a structured critical narrative strategy rather than duplicate systematic screening and was designed to compare translational patterns across diseases. It does not provide an exhaustive meta-analysis of every botanical intervention. Quantitative data were restricted to outcomes traceable to pivotal trials or established meta-analyses; when exact event counts or effect estimates were not secure, results were described qualitatively rather than reconstructed.

Study selection, data extraction and adjudication were carried out jointly by both authors rather than by independent duplicate screening, and the review used no protocol registration, formal risk-of-bias instrument or GRADE certainty rating. Quantitative values and safety conclusions were checked against the source publications and disagreements were resolved by discussion. All reported estimates are source-reported; no inferential statistic was recalculated by the authors, and the review is not registered or reported against PRISMA. Study omission and interpretive bias therefore remain possible. The evidence base is heterogeneous in product nomenclature, manufacturing disclosure, outcome definition and language; publication bias is likely, and most trials are underpowered for uncommon adverse events and treatment-effect heterogeneity.

Sex, gender, age, ancestry, comorbidity and polypharmacy were reported inconsistently, limiting subgroup inference and generalizability. The 2020–2026 literature was prioritized, but older pivotal studies were necessary because contemporary reviews often rest on the same small trials. Guideline and regulatory positions are living documents and should be rechecked immediately before submission.

Mechanistic coverage was intentionally selective. The review focused on pathways that could be related to characterized human products, plausible exposure and clinical endpoints; it did not catalog every reported molecular target. This approach reduces apparent breadth but improves falsifiability. Emerging organoid, single-cell and spatial data will require a future update when they are linked to clinically characterized preparations and exposure-compatible target engagement.

## 11. Conclusions

Multi-target natural products can provide adjunctive pharmacology, but the category does not carry a presumption of efficacy or safety. The most credible examples are product- and indication-specific: curcumin with mesalamine in mild-to-moderate UC, PA-free butterbur or *Nigella* oil as research-stage respiratory adjuncts, berberine for short-term metabolic surrogate improvement, and saffron for short-term depressive symptoms. Each remains bounded by limitations in sample size, standardization, duration or outcome maturity.

The strongest cautionary examples are equally informative. Indigo naturalis combines a compelling ulcerative-colitis signal with PAH risk; St John’s wort combines extract-dependent antidepressant efficacy with potent enzyme and transporter induction; and bioavailability enhancers can alter both desired exposure and interaction liability. These are not exceptions to natural-product pharmacology; they are central expressions of it.

A defensible development pathway is therefore evidence-gated: authenticate and quantify the product, establish human exposure, demonstrate concentration-compatible target engagement, test incremental benefit on validated endpoints, and define interaction and organ-toxicity boundaries. For IJMS readers, the central translational test is whether a chemically defined natural product achieves human exposure compatible with the claimed molecular target and whether target engagement is supported by disease-proximal biomarkers rather than retrospective pathway mapping alone. Under these conditions, selected natural products may complement modern pharmacotherapy. Without them, multi-target language risks becoming a substitute for pharmacological evidence.

## Data Availability

No new data were created or analyzed in this study. Data sharing is not applicable to this article.

## Abbreviations

ACT, Asthma Control Test; AhR, aryl hydrocarbon receptor; AMPK, AMP-activated protein kinase; CYP, cytochrome P450; FEV1, forced expiratory volume in 1 s; FPG, fasting plasma glucose; HAM-D, Hamilton Depression Rating Scale; IBD, inflammatory bowel disease; ICS, inhaled corticosteroid; PA, pyrrolizidine alkaloid; PAH, pulmonary arterial hypertension; P-gp, P-glycoprotein; RCT, randomized controlled trial; SSRI, selective serotonin reuptake inhibitor; TNSS, total nasal symptom score; UC, ulcerative colitis; UGT, uridine diphosphate glucuronosyltransferase.
